# Establishment of an Animal Model of Dog Bite Injuries

**DOI:** 10.7150/ijms.94432

**Published:** 2024-03-31

**Authors:** Dou Huang, Wenhao Jia, Kaide Li, Zhiru Liu, Lei Liu

**Affiliations:** State Key Laboratory of Oral Diseases & National Clinical Research Center for Oral Diseases & Department of Oral and Maxillofacial Surgery, West China Hospital of Stomatology, Sichuan University, Chengdu 610041, Sichuan Province, China.

**Keywords:** bites, dog, animal models, wounds and Injuries, sutures

## Abstract

Nowadays dog bite is becoming a world public health problem. Therefore, the study aimed to develop a dog bite animal model that is helpful to solve these problems. In this study, the skull of an adult dog was scanned. The three-dimensional model of the dog maxillofacial bones and dentition was built by MIMICS. Next, the model was printed with Co-Cr alloy by using selective laser sintering technology to develop the dog bite simulation pliers. Then, to simulate dog bite to most, the maximum bite force of the pliers was measured and actions contained in dog bite process was analyzed. Afterwards, according to action analysis results, rabbits were bitten by the prepared instrument in actions that simulate dog's bite. Finally, the reproducibility and controllability of this animal model of dog bite injuries was validated in an *in vivo* study. The results showed a reliable animal model of dog bite injuries has been developed in this study. The sites and severities of the injuries could be adjusted as the operator wishes and the animal model of dog bite injuries was highly repeatable. This study also indicates the feasibility of using digital technology in establishing animal bite models.

## Introduction

With the increasing number of dogs in modern society, the occurrence of dog bite injuries has become more frequent 1-3. According to the World Health Organization 4, dog bites are among the top 12 causes of non-fatal injuries worldwide. A dog bite can result in a severe laceration, avulsed tissue, and even bone fractures due to the dog's powerful occlusal force and sharp teeth 5-7. Furthermore, a dog's mouth is a source of pathogenic bacteria, which carries a risk of infection and rabies 8-10. In addition, the dog may hold on for a long duration of time to tear the victim's tissue. Damage caused by such prolonged bites raises the possibility of local tissue necrosis and avulsion, eventually resulting in tissue defects that severely impair patients' quality of life 11,12.

The current basic therapeutic principles of dog bite injuries have been established after long clinical practice 13,14. However, the specific treatments of dog bite injuries remain controversial 15-17. The reason lies in the complexity of the lesions in dog bite injuries, which often vary greatly in extent and depth, presents significant challenges in their treatment, such as the need for plastic sutures and the consideration of flap surgeries 18-19. Notably, all previous studies on dog bite injuries were clinical studies, and most of these were case reports or summaries of clinical experience; therefore, they lack adequate reliability 20-30. The main reason for this situation is that dog bite injuries range widely in severity and there are great individual differences among patients. Hence, a reproducible and comparable animal model is very meaningful and demanded.

The objective of this study was to establish an animal model of dog bite injuries by utilizing computer image processing and selective laser sintering technologies. We hypothesized that the dog bite simulation pliers could accurately replicate jaws with correct occlusal contact. The specific aims of the study were to develop the dog bite simulation pliers and to validate the efficacy in producing dog bite injuries.

## Materials and Methods

### Ethics statement

All animal experiment protocols were prospectively approved by the Research Ethics Committee for Laboratory Animal Science of West China Hospital of Stomatology, Sichuan University (Protocol Number: WCHSIRB-D-2017-251). This study was conducted in strict accordance with the recommendations of the Guide for the Care and Use of Laboratory Animals of the National Institutes of Health. Animals were housed in a temperature-, humidity-, and air renewal-controlled room. Animals were fed standard dried diet and water. All surgeries were performed under pentobarbital sodium anesthesia (30 mg/kg), and all efforts were made to minimize suffering.

### Data collection and analysis

A Chinese male rural dog with a correct bite and full dentition (age, 34 months; weight, 18 kg) was anesthetized with 30 mg/kg of pentobarbital sodium. The skull of the dog was then scanned by spiral computed tomography (CT) (Philips, the Netherlands). A three-dimensional model of the dog's maxillofacial bones and dentition was built from the spiral CT data by MIMICS software, version 16.0.0 (Materialise NV, Leuven, Belgium) and stored in standard template library format after modification.

### Preparation of dog bite simulation instrument

Cobalt-chromium (Co-Cr) alloy (Concept Laser, Lichtenfels, Germany) was used as the printing material 31. Through selective laser sintering technology, a direct metal laser sintering machine (EOSINT M 270; EOS, Krailling, Germany) was used to reconstruct a metallic model of the dog's maxillofacial bones and dentition. The model was welded on the end of the short handle of a pair of pliers to simulate jaws after correct occlusal contact had been established. The jaws were then sharpened to imitate the dog's teeth. The finished instrument was termed dog bite simulation pliers.

### Maximum bite force measurements of dog bite simulation instrument

The dog bite simulation instrument was used to bite a piezoelectric isometric Kistler force transducer (9311B, range ± 5000 N; Kistler Instrument Corp, Winterthur, Switzerland) with as much force as possible. The trial was repeated three times. Bite force data were recorded.

### Video analysis of dog bite action

To simulate a dog bite as accurately as possible, a hunk of flesh was used to induce a dog bite. The process was recorded by a video camera. Video camera captured views of the dog bite movements. Frame-by-frame video analysis was then used to document the timing and magnitude of behavioral events.

### Establishment of the new animal model of dog bite injuries

Four adult New Zealand white rabbits of either gender (age, 20-22 weeks; weight, 2-3 kg) were anesthetized with 30 mg/kg of pentobarbital sodium injected into the external ear vein. To simulate actual dog oral environment, the dog's saliva was collected by setting food before an adult dog to stimulate the production of saliva in this study. The collected saliva was about 10 ml and stored in a clean container. In order to ensure the quality of the saliva and avoid the overdue failure, the saliva was then coated to the jaws of dog bite simulation pliers as soon as possible before the *in vivo* experiment starts 32. One researcher simulated the process of a dog biting a rabbit with the dog bite simulation pliers according to the action analysis results. The type and severity of wounds caused by the dog bite simulation pliers were evaluated by the observation. Euthanasia of rabbits was induced by pentobarbital lethal injection.

### *In vivo* study

Twenty adult New Zealand white rabbits of either gender (age, 20-22 weeks; weight, 2-3 kg) were used to validate the reproducibility of the animal model of dog bite injuries. The rabbits were anesthetized with 30 mg/kg of pentobarbital sodium (Nembutal; Bayer, Leverkusen, Germany) injected into the external ear vein and then bit with the dog bite simulation pliers based the above method. Avulsed wounds about 3cm in length and deep to muscles were created on both hind limbs of rabbits. The wounds on the left limb received plastic surgery repair technique by suturing wounds in layers with 4-0 absorbable sutures, and then skin suturing with 5-0 silk sutures in the needle distance of 0.3-0.5cm. The wounds on the right limb received traditional repair technique by suturing wounds directly with 3-0 silk sutures in the needle distance of 1cm. A rubber drainage was put in the wounds and removed at 24 hours after operation. After surgery, the rabbits were given cefaclor (HuaBei Pharma, Hebei, China) at a dose of 20 mg/kg for 3 days. For analgesia, animals were dosed with subcutaneous injection of buprenorphine hydrochloride (0.03 mg/kg; Institute of Pharmaceutical Research, Tianjin, China) once a day for three days. After surgery, animals were housed at suitable room temperature and relative humidity. The wounds were examined daily for 7 days before the sutures were removed. The presence of infection in both groups was recorded. At the end of the fourth week after sutures out, the morphology of the scars was observed. The pigmentation, pliability, and height of the scars were recorded and assessed by a Modified Vancouver Scar Scale (MVSS) 33. Euthanasia of rabbits was induced by pentobarbital lethal injection.

### Statistical Analysis

All quantitative data was expressed as the mean ± standard deviation. For comparison of two groups, statistical analyses were performed using Fisher exact probability test and Kruskal-Wallis test in SPSS 22.0 software (Chicago, Illinois, United States) for Windows (version 18.0.0). A value of P<0.05 was considered to be statistically significant.

## Results

### Design and production of dog bite simulation pliers

**Figure 1:**
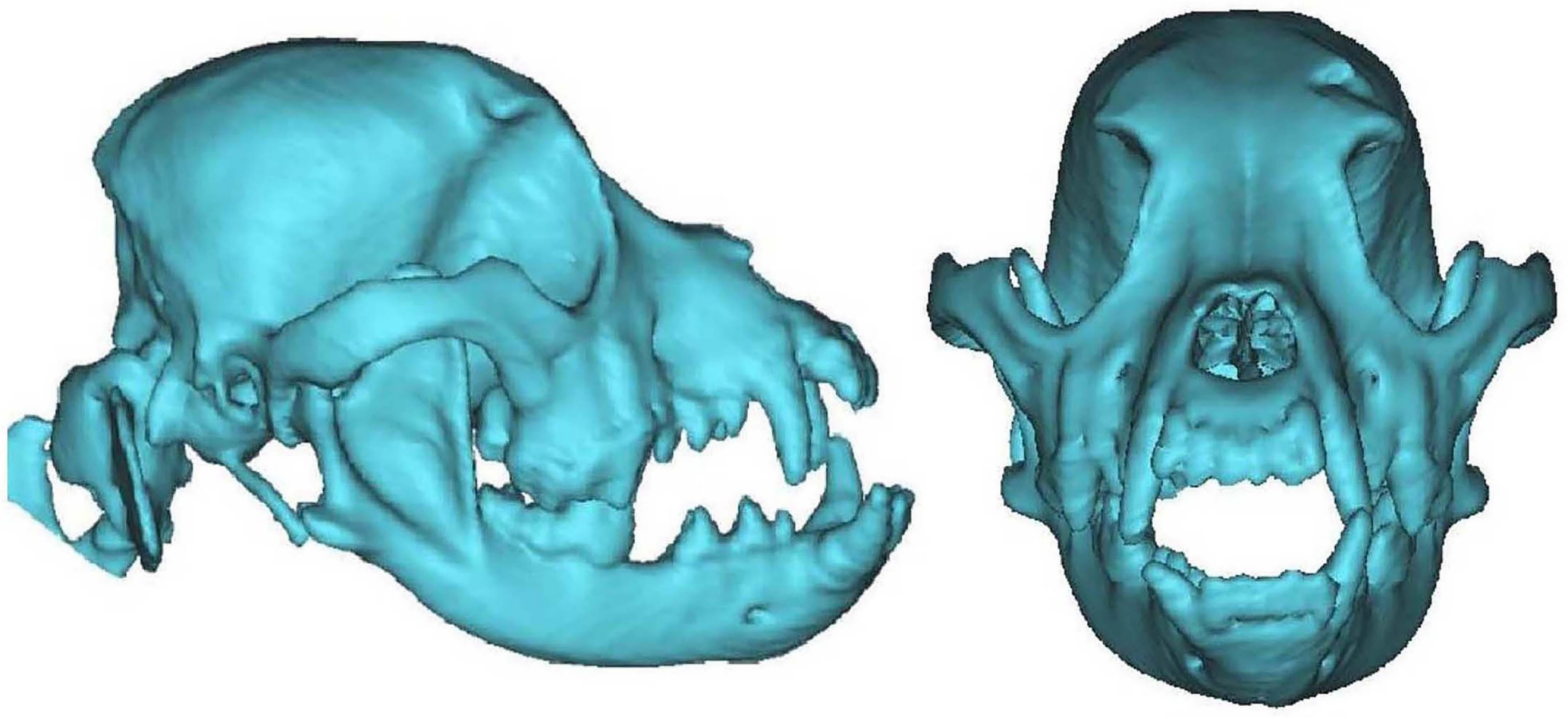
** Three-dimensional digital model of maxillofacial bones and dentition.** The images of the maxillofacial bones and dentition of Chinese rural dog were obtained with Mimics16.0.

**Figure 2:**
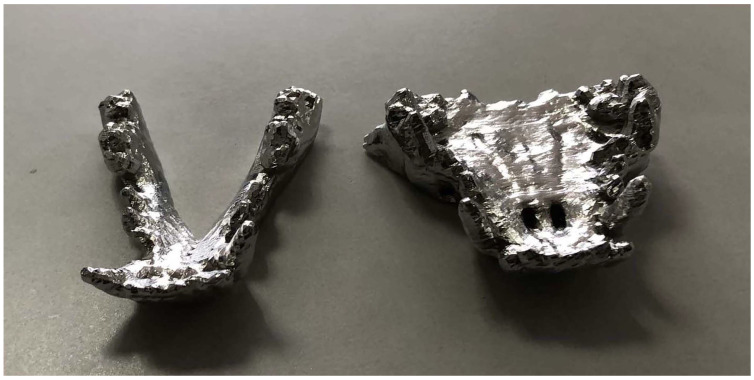
** The jaws of the dog bite simulation pliers.** Co-Cr alloy jaw model obtained by 3D printing.

**Figure 3:**
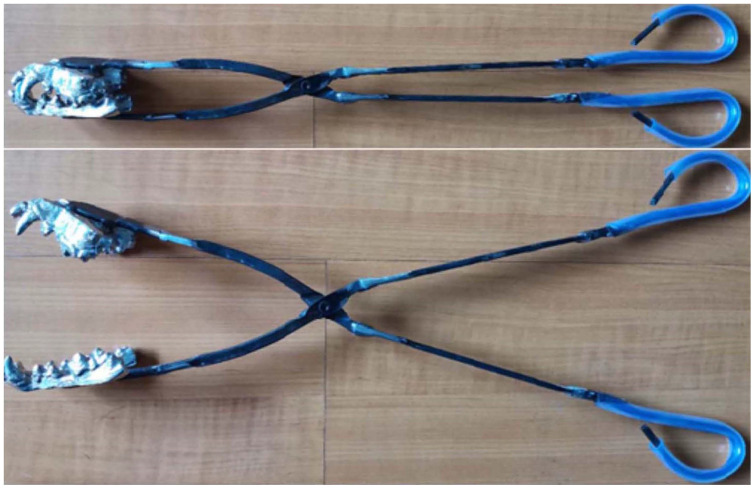
** The dog bite simulation pliers.** The model was welded on the end of the short handle of a pair of pliers to simulate jaws with correct occlusal contact.

The three-dimensional model of the dog's maxillofacial bones and dentition was reconstructed by MIMICS software (Figure 1). The metallic model was printed with Co-Cr alloy as the jaws of the dog bite simulation pliers. The jaws were consistent with the structure and size of the dog's maxillofacial bones and dentition and exhibited a normal occlusal relationship (Figure 2). The finished dog bite simulation pliers were able to simulate the opening and closing of the dog's mouth (Figure 3).

### Bite force measurement results of the dog bite simulation pliers

The bite force measured in the study was found to range from 1549 to 1601 Newtons (N), with a mean value of 1579 N (trial 1: 1587N, trial 2:1601 N, trial 3: 1549N).

### Dog bite action analysis

Using the video camera evidence, we found that the dog bite was a prolonged and complicated process starting with the dog's sharp teeth puncturing the flesh. Occlusal pressure was then applied as the dog held the flesh in its mouth. The dog shook its head and stepped back while biting to twist, tear, and drag the flesh.

### Establishment of the new animal model of dog bite injuries

**Figure 4:**
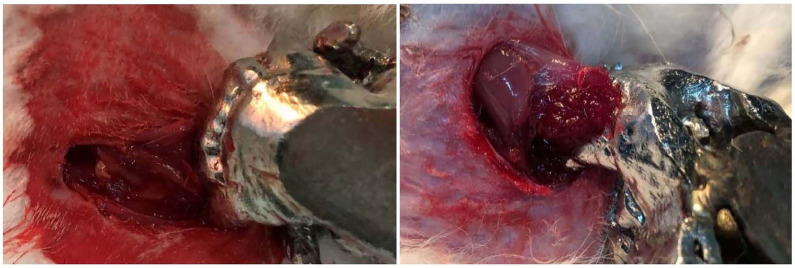
** The wounds made with the dog bite simulation pliers.** The skin and subcutaneous fascia and the muscle tissue of the four rabbits were torn off by the dog bite simulation pliers.

The tissues of the four rabbits, including the skin, blood vessels, and muscles, were easily punctured or ripped by the dog bite simulation pliers (Figure 4). Different types of injuries, including penetration, laceration and avulsion, were made on the rabbits in actions that simulated a dog bite. All the injuries caused by the pliers were similar to dog bite injures 34. All potential sites, particularly the craniofacial region which is the most frequently affected area 35, could be selected randomly. The severities of the injuries could be adjusted as the executor wishes.

### *In vivo* study

**Figure 5:**
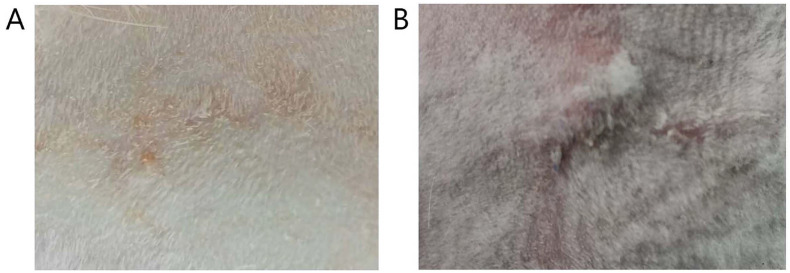
** The morphology of the scars.** Nineteen of the twenty rabbits were involved. (A) Scar of wound sutured by plastic surgery repair technique. (B) Scar of wound sutured by traditional repair technique.

The avulsed wounds were successfully established on nineteen of the twenty rabbits except one rabbit died during induction of anaesthesia. The results showed that all the wounds were made in same type and severity as requested, 3cm in length and deep to muscles. The infection rate of plastic surgery repair technique group was lower than that of traditional repair technique group, but without significant difference (P>0.05) (Table 1). The observation on scar appearance showed that scar tissues in plastic surgery repair technique group were generally smaller and paler compared with that in traditional repair technique group (Figure 5). According to the assessment results of MVSS, the score of scars in plastic surgery repair technique group was lower than that in traditional repair technique group. The difference was statistically significant (P<0.05) (Table 1).

## Discussion

Nowadays, dog bites have become a global public health issue 11,12,36,37. However, the specific treatments of dog bite injuries remain controversial 15-17. Animal experiments can provide important references for clinical research and practice, and establishment of an animal model is the first key step in performing an animal experiment. Therefore, this study was designed to establish an animal model of dog bite injuries.

In some previous work, the authors proposed using experimental dogs to bite laboratory animals to create an animal model of dog bite injuries. However, it is difficult to control the severity, site, and type of injuries in such animals, in turn making it difficult to conduct a randomized controlled trial in this manner. Moreover, this method leads to high mortality among the animals bitten by the dogs, hindering the establishment of an animal model. Therefore, we designed a new type of instrument that simulates the dog bite process, expecting that this instrument would be helpful in the establishment of an animal model of dog bite injuries. A challenging aspect of this idea is that it is difficult to reconstruct a dog's complicated dentition and maxillofacial bone structure as a model. Fortunately, with the development of computer image processing and selective laser sintering technologies 37,38, it has become possible to reconstruct an accurate simulation model of the dog's maxillofacial bones and dentition.

In this study, the dog involved was a Chinese rural dog. According to the publications, Chinese rural dogs have been blamed for many dog bite cases every year 39. The data of the dog's skull was obtained by spiral CT scans. A three-dimensional model of the maxillofacial bones and dentition was then built by MIMICS software. The metallic model was printed with Co-Cr alloy by selective laser sintering technology. Therefore, the dog bite simulation pliers developed in the study had highly similarities with the dog's oral and maxillofacial structure. Then, the bite forces of the dog bite simulation pliers were measured. It was found that the bite force of the pliers was strong enough compared to the jaw force that a dog normally had, which was found to range from 13 to 3417 N in previous studies 40,41. The results suggested that forces provided by the dog bite simulation pliers could match dog bites in strength.

The actions involved in the dog bite process were analyzed and simulated during use of the dog bite simulation pliers. Due to the fact that a large variety of bacteria can be found in a dog's mouth, dog saliva was collected and coated onto the jaws of the new instrument to simulate the actual oral environment of a dog as accurately as possible. Based on these, the animal model of dog bite injuries was established on rabbits with the use of the dog bite simulation pliers. The results showed that the jaws of the pliers were strong and sharp enough to tear rabbits' tissues. As a result, these dog bite simulation pliers could easily be used to create injuries of various severities in rabbits. Additionally, the animal model of dog bite injuries established in this study was highly controllable and totally determined by the controller. More importantly, the injuries were quite similar to dog bites. Finally, the reproducibility of this animal model was validated in the *in vivo* study. It was found that all the wounds were made in same type and severity on different individual rabbits, indicating that this animal model could be replicated well. Consequently, a new reliable and controllable animal model of dog bite injuries was developed in this study.

The results of the *in vivo* study also showed that plastic surgery repair technique successfully minimized scarring without increasing the risk of infection, compared to traditional repair technique. These results suggested that plastic surgery repair technique may be a better option in the treatment of dog bite injuries.

In this study, computer image processing technology was used in combination with selective laser sintering technology to develop dog bite simulation pliers for the first time. The use of these digital technologies achieves highly accurate simulation, good controllability, and high convenience required in an animal model of dog bite injuries. This study demonstrated that digital technologies can assist in the establishment of animal models of dog bite injuries, indicating that the method has the potential to be applied to similar research, especially studies involving animal bite models. Although the current study design might be imperfect, it presents an important first step towards such further researches. It will stimulate the interest of other researchers to focus on modification of the animal model of dog bite injuries, and optimal treatment plans.

## Conclusions

In conclusion, we have established a new and reliable animal model that may help in further studies on dog bite injuries.

## Figures and Tables

**Table 1:** Infection rate and MVSS score of wounds with two different sutures.

	Plastic surgery repair technique	Traditional repair technique	P values
**Infection (n, %)**	3(15.78%)	4 (21.05%)	p > 0.05
**No infection (n,%)**	16(84.22%)	15 (78.95%)	
**MVSS-Sum (means ± standard)**	5.26 ± 1.12	8.32 ± 1.84	p < 0.05

Note: MVSS-Sum: Sum of means of MVSS parameters.
